# Semen Evaluation from Dominant Males of the Viviparous Mexican Lizard *Sceloporus torquatus*, Wiegmann, 1828 (Sauria: Phrynosomatidae)

**DOI:** 10.3390/vetsci12040363

**Published:** 2025-04-13

**Authors:** Martín Martínez-Torres, Uriel Ángel Sánchez-Rivera, Alfredo Medrano, Enrique Othón Hernández-Gónzalez, Rodrigo Dávila-Govantes, Yabín Josué Castro-Camacho, Norma Berenice Cruz-Cano

**Affiliations:** 1 Laboratorio de Investigación de Saurios en Asistencia Reproductiva y Desarrollo (LISARD), Unidad de Morfología y Función, Facultad de Estudios Superiores Iztacala, Universidad Nacional Autónoma de México, Av. De los Barrios # 1, Los Reyes Iztacala, Tlanepantla 54090, Estado de Mexico, Mexico; rodago95@gmail.com (R.D.-G.); yabin.castro@iztacala.unam.mx (Y.J.C.-C.); norma.cruz@iztacala.unam.mx (N.B.C.-C.); 2 Laboratorio de Reproducción Animal, Unidad de Investigación Multidiciplinaria, Facultad de Estudios Superiores Cuautitlán, Universidad Nacional Autónoma de México, Cuautitlán Izcalli 54714, Estado de Mexico, Mexico; amedrano@unam.mx; 3 Departamento de Biología Celular, Centro de Investigación y Estudios Avanzados, Instituto Politécnico Nacional, Av. Instituto Politécnico Nacional 2508, San Pedro Zacatenco, Alcaldía Gustavo A. Madero, Ciudad de Mexico 07360, Mexico; enrique.hernandez@cinvestav.mx

**Keywords:** reptiles, assisted reproduction, spermatozoa, fertility assessment, male selection

## Abstract

While most lizard species are polygamous, dominant males typically have greater access to females and copulate more frequently than submissive ones. Several studies suggest that applying assisted reproduction techniques to this taxon could be an effective tool to enhance conservation programs. Therefore, the proper selection of males for assisted breeding programs is crucial for their overall success. In this study, we report for the first time data on semen and sperm quality from dominant males of the Mexican lizard *Sceloporus torquatus* using techniques commonly employed in assessing mammalian fertility. The semen and sperm characteristics were as follows: volume, 14.0 µL; sperm concentration, 125.7 × 10^6^ cells/mL; wave motion, 2.7; total motility, 87.8%; sperm viability, 89.0%; normal morphology, 88.8%; plasma membrane integrity, 87.7%; low plasma membrane fluidity, 94.9%; capacitation status: uncapacitated (F), 90.5%; capacitated (B), 7.2%; sperm with acrosome reaction (AR), 2.3%; and, acrosome integrity, 88.8%. These results suggest that, according to mammalian fertility parameters, dominant males of *S. torquatus* are fertile. This series of tests provides a valuable tool for conducting a comprehensive analysis of the functional changes that may occur in sperm handling during assisted processes, and that may contribute—along with the proper selection of individuals—to reproductive success.

## Simple Summary

In many lizard populations, males are polygamous; however, dominant males have greater access to females and more opportunities to copulate. It is important to conduct a seminal evaluation as this will help determine the reproductive potential of the organisms and assess their suitability for assisted reproduction methods. In this study, we report for the first time data on the semen and sperm quality from dominant males of the Mexican lizard *Sceloporus torquatus* using techniques commonly employed to assess mammalian fertility. This series of evaluations allowed us to perform a comprehensive analysis of seminal characteristics, providing a deeper understanding of sperm functionality. Such assessments may play a significant role in the success of assisted reproduction for this species.

## 1. Introduction

Reproduction is essential for the survival of any species, which is why sexually reproducing organisms have developed various strategies to find a mate and ensure reproductive success. Competition among lizards to copulate with females has led to the evolution of traits that enhance their chances of winning agonistic encounters with other males, such as larger body size, greater biting force, or specialized weapons [1]. Additionally, postcopulatory sexual selection plays a crucial role in the reproductive process [2]. In natural populations, individuals differ in their ability to survive and reproduce, leading to variations in the number of gene copies they pass on to the next generation [3]. While most lizard species are polygamous, dominant males generally have greater access to females and copulate more frequently than submissive males [4]. Consequently, their reproductive success is primarily determined by the number of females they can inseminate. Male fertility has rarely been studied in natural populations as it was previously believed that strong selection would result in uniformly high fertility levels among males, leading to the assumption that mating success equates to fertilization success [3]. Thus, pregnancy rates in natural lizard populations depend on both the innate fertility of males and oocyte quality.

On the other hand, the decline in herpetofauna is a global phenomenon, and numerous studies have documented a dramatic reduction in populations of various saurian species worldwide [5,6,7,8]. Additionally, it has been reported that one-fifth of lizard species are currently under some threat category [9]. Several authors agree that applying assisted reproduction methodologies in this taxon could be an excellent tool to enhance conservation programs [10,11,12].

Several non-destructive methods have recently been implemented to obtain semen from lizards [11,12,13]. These techniques allow for the collection of multiple ejaculates from each male to assess sperm quality before assigning them to any assisted reproduction trials. The proper selection of males for use in assisted reproduction programs is a critical factor in the overall success of these programs. However, there have been limited efforts to understand which semen traits determine male fertility in natural populations, including reptiles. Therefore, we believe it is essential to conduct semen evaluations of dominant males to establish reference parameters for wild lizard populations.

We selected *Sceloporus torquatus* as our model species because, in addition to being classified as a species of least concern by the IUCN in 2020 [14], there is extensive knowledge about its male reproductive biology. This includes information on its reproductive cycle [15], changes in testicular cytology, and testosterone levels during the reproductive cycle [16], semen collection [11,13], seasonal mating, sperm storage [17], and sperm cryopreservation [18,19]. This knowledge has been instrumental in interpreting the results obtained in our study. *S. torquatus* is a robust lizard species that is abundant and widely distributed across several states in the Central Plateau of Mexico, including Veracruz, Hidalgo, Estado de México, Mexico City, Puebla, and Morelos [20]. Its altitudinal distribution ranges from 1400 to 2800 m above sea level [21]. This species exhibits asynchronous reproductive cycles between males and females, with spermatogenesis beginning in May, testicular mass and volume reaching their maximum size in September [16], and mating occurring in October, several weeks before ovulation, necessitating obligatory sperm storage in the vagina [17]. In this study, we evaluated semen and sperm quality from dominant males in a wild population of the Mexican lizard *S. torquatus* using a set of tests commonly employed for fertility evaluation in mammals. We found that dominant males exhibited elevated values in all sperm quality parameters evaluated.

## 2. Materials and Methods

### 2.1. Organisms

Adult males (*n* = 23) and females (*n* = 11) of *S. torquatus* were collected during the first week of October in 2020 and 2022. Males had an average snout-vent length of 94 ± 3.9 mm and an average weight of 32.8 ± 4.40 g, while females had an average snout-vent length of 92.2 ± 6.2 mm and an average weight of 29.9 ± 5.5 g. These specimens were collected in Sierra de Guadalupe State Park (19°37′–19°29′ N; 99°12′–99°02′ W), at altitudes ranging from 2200 to 3000 m, just before the mating season (last week of October) [17,18]. The collection was carried out under the scientific collecting licenses (SGPA/DGVS/02921/19 and SGPA/DGVS/08681/21) granted by the Secretaría del Medio Ambiente y Recursos Naturales. On the same day of capture, the animals were toe-clipped for individual identification, and their snout-vent length and body weight were recorded. The following day, males and females were released into separate terrariums (2.5 m × 2.5 m × 5.0 m) located inside the greenhouse of the Facultad de Estudios Superiores Iztacala (19°52′59″ N, 9°18′73″ W, 2240 m altitude). These terrariums were equipped with rocks and trunks to mimic their natural habitat and were maintained at a temperature and photoperiod corresponding to natural conditions. The animals had free access to water and were provided with food—including mealworms, grasshoppers, and crickets—throughout the entire experimental period.

### 2.2. Male Selection

The collected males underwent a courtship test to select individuals for sperm assessment. A video camera was placed inside a terrarium (2.5 m × 2.5 m × 5.0 m) to record the lizards’ behavior. Subsequently, three males, each marked with vegetable paint for easy identification, were introduced into the terrarium. Thirty minutes later, two to three females were also introduced, and their interactions were recorded on video for a period of 30 to 60 min. Only males exhibiting dominant and courtship behaviors—such as male–male confrontations, displacement of rival males, nodding, “shuddering”, running around, or attempting to mount the females [4,22,23]—were separated from the group and placed individually in another terrarium.

### 2.3. Semen Collection

The selected males were anesthetized through intraperitoneal administration of pentobarbital sodium (16.0 µg/10 g body weight) [13]. Once the lizards reached a deep state of relaxation, a thorough surgical cleaning was performed to remove excreta and urate residues. This involved cleaning the body with a 3% sodium hypochlorite solution for hygiene purposes and cleansing the cloaca with a reptile saline solution (0.7% NaCl *w*/*v*) [24]. Subsequently, semen was extracted by applying gentle pressure to the genital papilla, following the method described by Martínez-Torres et al. [11]. This procedure was conducted under a stereoscopic microscope and was repeated up to four times for each male.

### 2.4. Semen Assessment

Immediately after collection, semen volume was measured using purpose-calibrated pipette tips (at 1.0 µL intervals). The semen was then transferred to 0.5 mL plastic tubes and placed in a water bath at 24 °C. Without delay, the wave motion of the neat semen was evaluated under light microscopy (Leica DMLS, Heerbrugg, Switzerland) using a 10× objective. A subjective scale ranging from 0 to 3 was employed to assess wave motion (0 = no movement, 3 = fast and dense waves), following the approach of González-Urdiales et al. [25].

The remaining semen was diluted to 200 μL with Tyrode’s medium [26] at 24 °C and allowed to adapt to the new medium for 10 min before assessment. Sperm concentration was estimated by counting spermatozoa in a Neubauer [27] chamber using a 1:200 dilution (semen–formaldehyde saline solution) [26]. Total motility, defined as the percentage of sperm displaying movement, was subjectively evaluated using a slide and coverslip under light microscopy (magnification 100×). Sperm viability, distinguishing between live (unstained) and dead (stained) cells, and morphology (normal, primary, or secondary abnormalities) were assessed using eosin/nigrosine staining under light microscopy (magnification 1000×) following the criteria established for mammalian spermatozoa [26,28].

Plasma membrane integrity was evaluated using SYBR14/PI fluorescent stain [29] under fluorescent microscopy (Leica DMLS, magnification is 1000×). Green fluorescence across the head indicated intact plasma membrane spermatozoa, while red fluorescence on part or all of the head indicated damaged plasma membrane spermatozoa.

Plasma membrane fluidity was assessed using Merocianine 540 (MC540, Zigma Chemical) following a protocol developed for pigs [30,31] under fluorescent microscopy with a magnification of 1000×. Dull (opaque) fluorescence across the head indicates low plasma membrane fluidity, while brilliant fluorescence indicates high plasma membrane fluidity.

Although sperm capacitation was not previously demonstrated in reptiles, an assay to assess capacitation status was conducted using the chlortetracycline test (CTC) under fluorescent microscopy (magnification 1000×) following a protocol developed for pig spermatozoa [32]. After the assay, spermatozoa were classified into three patterns: pattern F (non-capacitated, acrosome-intact), displaying uniform fluorescence over the entire head; pattern B (capacitated, acrosome-intact), showing a fluorescence-free band in the post-acrosomal region; and the acrosome-reacted (AR) pattern, displaying either a fluorescence band in the equatorial segment or no fluorescence. Lastly, acrosome integrity was evaluated using *Pisum sativum* fluorescent lectins, as described by Medrano et al. [33]. After the assay, sperm were categorized as either acrosome-intact or acrosome-damaged.

## 3. Results

Semen was successfully obtained from all “dominant males” (*n* = 16). It exhibited a milky-white appearance with a viscous consistency, and the values for each test are presented in Table 1.

**Figure 1:**
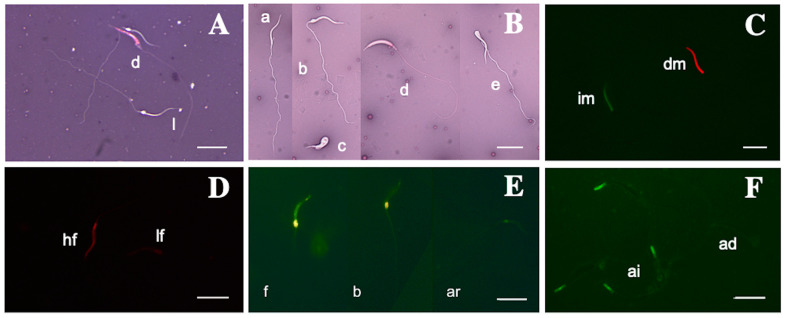
Sperm quality analysis of “dominant males” of *Sceloporus torquatus*: (**A**) Eosin–Nigrosin staining for live (l) and dead (d) sperm; (**B**) normal (a), cytoplasmic droplet (b), abnormal development (c), swollen head (d), and bent head (e); (**C**) SYBR14/Propidium Iodide staining for intact (im) and damaged (dm) membranes; (**D**) Merocyanine staining for sperm with high fluidity (hf) and low fluidity (lf); (**E**) chlortetracycline staining for full (f), banded (b), or reacted (ar) sperm; (**F**) fluorescent lectin staining to reveal acrosome-intact (ai) or acrosome-damaged (ad) sperm. Scale bars correspond to 10 µm.

The semen volume showed significant variability, ranging from 7.0 to 27.0 μL. Sperm concentration ranged from 51.1 to 179.3 × 10^6^ cells/mL, with an average of 125.7 ± 62.2 × 10^6^ cells. Similarly, both the wave motion and total motility varied, with values of 2.7 ± 0.4 and 87.8 ± 9.8%, respectively. Furthermore, the viability averaged 89.0 ± 7.2% (Figure 1A); normal morphology of the sperm was found to be 88.8 ± 8.5%, with primary and secondary abnormalities at 5.5 ± 4.8% and 5.6 ± 5.0%, respectively (Figure 1B); and plasma membrane integrity was consistent at 87.7 ± 8.1% (Figure 1C). The proportions of spermatozoa displaying either low or high plasma membrane fluidity were 94.9 ± 2.9% and 5.0 ± 2.9%, respectively (Figure 1D). The capacitation status was as follows in the CTC assay: 90.5 ± 5.2% for pattern F, 7.7 ± 5.4% for pattern B, and 2.3 ± 1.3% for pattern AR (Figure 1E). Regarding the acrosome integrity, 88.8 ± 7.7% remained intact, while the rest was damaged (Figure 1F).

## 4. Discussion

### 4.1. Dominant Male Characteristics

In lizards, dominant males are primarily responsible for executing aggressive behaviors within the group, such as displays, courtship, fights, chases, and/or mating behaviors [22]. Notably, male–male aggressive interactions are strongly correlated with body size and dominance [34,35,36,37,38]. In our study, dominant males were identified as those exceeding 90 mm in snout-vent length and exhibited courtship, mating, and/or victorious behaviors during male–male confrontations. These dominant males actively prevent smaller males from accessing females. Currently, no established reference values exist for assessing sperm quality in reptiles; however, the values recorded for these dominant males suggest that, based on fertility parameters established for mammals, the spermatozoa of *S. torquatus* “dominant males” exhibit high quality, classifying them as fertile individuals [26].

### 4.2. Semen Characteristics

While lizards employ various strategies to achieve reproductive success [39], sperm quality plays a pivotal role in attaining it. Given the alarming decline in the populations of numerous lizard species worldwide [9], it is crucial to assess sperm quality before utilizing individuals for assisted reproduction methods. The observed semen color and consistency align with previous reports for this species [11,13], as well as those for other saurian species, such as *Iguana iguana* [40], *S. aeneus*, *S. grammicus*, and *Phrynosoma orbiculare* [11].

Previous research has suggested a correlation between semen color and consistency with sperm concentration [40]. Our assays did not reveal any significant alterations in these characteristics. Instead, we observed that the seminal volume exceeded previously documented levels for this species [13]. This may be because we captured the males before the mating season and obtained the samples at the beginning of this period. Nevertheless, we do not know if the quality or consistency of semen decreases toward the end of the mating season.

On the other hand, although the exact amount of semen deposited during each copulation remains unknown, males with larger semen volumes may have a heightened opportunity to mate with multiple females or engage in more copulations with the same female. If this is true, then this situation increases the likelihood that males with greater semen volume will produce more offspring per litter or have offspring with multiple females. Moreover, larger semen volumes may allow for multiple trials and facilitate the development of assisted reproduction methods.

### 4.3. Sperm Characteristics

Regarding sperm concentration, sperm motility, sperm viability, and the percentage of normal sperm, our results showed considerable variability. However, they were generally high and consistent with prior reports for this species [11,13]. A similar trend has been observed in other lizard species, including *I. iguana* [40], *Tropidurus spinulosus* [41], and *S. aeneus*, *S. grammicus*, *S. anahuacus*, and *Phrynosoma dugesi* [11]. Several factors may contribute to the observed variability in these parameters, including diet [42], temperature, photoperiod, and pollutants from the suburban environment in which these lizards reside [43,44], among others. The cytological evaluation of these abnormalities is valuable; however, its contribution to assessing sperm quality should be interpreted with caution. While variability is common among males, it remains uncertain whether any of these factors significantly influence male reproductive potential. Therefore, further research on this topic is essential as it could be a limiting factor in developing an effective assisted reproduction methodology. Although the motility values reported here are useful, subjective assessment lacks the precision to consider total motility a relevant indicator of fertility, making the incorporation of computer-assisted sperm analysis (CASA) beneficial for implementing assisted reproduction technologies in lizards. However, access to this tool may be limited in some contexts [45,46].

Considering that the fertilizing potential of sperm is intricately linked to its functional capabilities [47], attributes such as plasma membrane integrity, membrane fluidity, and acrosome integrity are critical for various spermatozoa activities. In mammals, these activities include processes like capacitation, acrosome reaction, and interaction with the zona pellucida [48,49]. We suggest that assessing these plasma membrane parameters should be integrated into routine seminal analyses of reptiles, as in mammals, these characteristics are valuable predictors of in vitro fertilization capacity [49,50]. Our tests revealed a substantial percentage of spermatozoa with intact plasma membranes and acrosomes, which, in mammals, are indicative of high sperm quality [51]. While reptile-specific data are lacking, we believe that, similar to mammals, these values could indicate good sperm quality in reptiles as well.

On another note, membrane fluidity and capacitation status have not yet been evaluated in fresh semen from any reptile species to date. We observed a notable percentage of spermatozoa displaying low plasma membrane fluidity. In mammals, membrane fluidity develops during sperm maturation in the epididymis and further increases during capacitation [52,53]. This increase is crucial for initiating signaling cascades that trigger the acrosomal reaction [53,54]. However, spermatozoa with high membrane fluidity are more susceptible to damage from reactive oxygen species [55]. It is well-established that membrane lipid peroxidation can challenge sperm survival and fertility [56,57]. Considering that *S. torquatus* spermatozoa must be stored in specialized tubules until ovulation occurs (approximately 4 weeks after copulation, Martínez-Torres, unpublished data), it is advantageous for them to exhibit low membrane fluidity. This enhances their chances of survival and preserves their fertility potential for an extended period, similar to mammals, where spermatozoa with rigid membranes delay the acrosomal reaction [58]. Furthermore, it has been observed that spermatozoa stored in the isthmus delay capacitation until ovulation-related signals induce their release, thereby maintaining fertility [59,60,61]. A similar scenario may occur in lizards during sperm retention in the vagina.

Numerous studies have demonstrated that the CTC assay is a useful tool for determining the capacitation status of sperm in several mammalian species (including humans, mice, and rabbits) [62,63,64,65]. CTC binds to membrane-associated cations—especially Ca^2+^—becoming more fluorescent, which results in three characteristic staining patterns exhibited by the sperm membranes across all mammalian species studied [63]. Although sperm capacitation in reptiles has been questioned [66], our findings, supported by the CTC test, provide evidence that sperm capacitation also occurs in reptiles, similar to its occurrence in mammals. Furthermore, we recently observed that after incubation in the BWW capacitating medium, there was an increase in the percentage of spermatozoa exhibiting pattern B as well as a change in flagellar movement similar to the “hyperactivated movement” seen in capacitated mammalian spermatozoa [67]. On the other hand, Nixon et al. [68,69] provided evidence that changes analogous to mammalian sperm capacitation occur in the spermatozoa of the Australian saltwater crocodile (*Crocodylus porosus*). They found that cyclic AMP levels were significantly elevated after incubation in BWW medium, which enhanced sperm motility and increased protein phosphorylation levels. Our findings are significant for two key reasons: (1) they suggest that sperm capacitation is a process established in reptiles and has been evolutionarily conserved in mammals; and (2) they suggest that, in saurians—like in mammals—changes occur during the journey through the female reproductive tract that promote sperm capacitation.

### 4.4. Perspectives

While the data presented here provide a valuable reference for the sperm quality profile of “dominant males” from a thriving endemic species, further tests are necessary to definitively assess the fertility potential of each male’s sperm. These additional tests may include evaluations such as sperm adhesion–penetration tests and paternity tests. Moreover, it is crucial to recognize that species-specific quality parameters conducive to the successful application of assisted reproduction programs need to be established. Notably, regardless of the species, data on sperm morphology (including sperm viability, normal morphology, and acrosome integrity percentages) as well as physiological aspects (such as sperm motility and membrane fluidity) serve as vital indicators of a male’s reproductive fitness in any reptile species.

## 5. Conclusions

Our study presents the initial findings regarding the quality assessment of semen from dominant lizards, employing conventional techniques commonly used to evaluate fertility in mammals. Our key conclusions are as follows: (1) Several fundamental characteristics—including volume, sperm concentration, wave and total motility, sperm viability, and normal morphology—exhibited notably high values and substantial variability among the male lizards examined. (2) These data strongly indicate that the reproductive health and general condition of the sampled individuals are quite favorable, aligning with established standards for humans and domestic mammals such as dogs, cats, and swine. (3) Notably, the high values observed in total motility, which is known to correlate with sperm viability, along with the functionality of the plasma membrane and acrosome status, which are linked to fertilizing capacity, suggest that males with spermatozoa displaying robust motility, viability, and normal morphology should be regarded as fertile individuals. (4) It is imperative to note that further investigations are essential involving males with confirmed fertility in order to establish reference values for semen and spermatozoa in other lizard species. This will enable the comprehensive evaluation of reproductive potential within wild lizard populations.

## Figures and Tables

**Table 1:** Seminal and spermatic characteristics of the Mexican viviparous lizard *Sceloporus torquatus*.

Characteristics		Mean ± SD	Interval
Ejaculates (*n*)		3.2 ± 0.8	2–4
Volume ^1^ (µL)		14.0 ± 5.4	7.0–27.0
Wave motility (1–3)		2.7 ± 0.4	2–4
Total motility (%)		87.8 ± 9.8	72.0–96.5
Sperm concentration (×10^6^ cells/mL)		125.7 ± 62.2	51.1–179.3
Sperm viability (%)		89.0 ± 7.2	72.0–96.5
Plasma membrane integrity (%)		87.7 ± 8.1	70.0–98.0
Plasma membrane fluidity (%)	Low	94.9 ± 2.9	89.0–98.0
	High	5.0 ± 2.9	2.0–11.0
Acrosome integrity (%)		88.8 ± 7.7	70.0–97.0
Sperm morphology (%)	Normal	88.8 ± 8.5	70.5–98.0
	Primary ^2^	5.5 ± 4.8	1–15
	Secondary ^2^	5.6 ± 5.0	1–17
Sperm capacitation status (%)	F	90.5 ± 5.2	80.0–98.0
	B	7.7 ± 5.4	1.0–19.0
	AR	2.2 ± 1.3	1.0–5.0

^1^ considering the total number of ejaculates per male. ^2^ abnormalities.

## Data Availability

The raw data supporting the conclusions of this article will be made available by the authors upon request.
